# Complete Genome Sequence of *Streptococcus anginosus* J4211, a Clinical Isolate

**DOI:** 10.1128/genomeA.01440-15

**Published:** 2015-12-17

**Authors:** Maliha Rahman, Scott V. Nguyen, Kimberly A. McCullor, Catherine J. King, James H. Jorgensen, W. Michael McShan

**Affiliations:** a Department of Pharmaceutical Sciences, The University of Oklahoma College of Pharmacy, Oklahoma City, Oklahoma, USA; b Departments of Pathology and Medicine, University of Texas Health Sciences Center at San Antonio, San Antonio, Texas, USA

## Abstract

*Streptococcus anginosus* is an opportunistic human pathogen that causes abscesses of the brain, liver, and other organs. Here, we announce the complete genome sequence of a clinically isolated strain of *S. anginosus* J4211. The genome sequence contains two prophages and multiple mobile genetic elements.

## GENOME ANNOUNCEMENT

*Streptococcus anginosus* belongs to the *S. anginosus* group (SAG), a subgroup of viridans streptococci. This group includes two other species, *Streptococcus intermedius* and *Streptococcus constellatus* (1, 2). These streptococci are typically commensal human flora of the oral cavity and gastrointestinal and genital tracts (3). However, they can occasionally cause opportunistic infections, particularly brain and liver abscesses, and are emerging pathogens in cystic fibrosis patients (4, 5). *S. anginosus* strain J4211 was originally a clinical isolate from the University of Texas Health Sciences Center at San Antonio, San Antonio, TX.

The strain was grown in Todd-Hewitt broth with 2% yeast extract (THY) broth supplemented with 5% heat-inactivated horse serum (THY-HS) overnight at 37°C. Chromosomal DNA was isolated as previously described (6, 7). The DNA library was prepared using the Nextera DNA library preparation kit (Illumina, Inc., San Diego, CA), and library quality was checked with an Agilent high-sensitivity DNA chip. Genome sequencing was performed by the Illumina MiSeq using paired-end 250-bp sequencing with high coverage (600-fold) at the University of Oklahoma Health Sciences Center, Laboratory for Genomics and Bioinformatics, Oklahoma City, OK. DNA assembly was done using the software package Geneious. Sequence gaps were closed by PCR amplification. Gene annotation was performed by the Rapid Annotations using Subsystems Technology (RAST) pipeline (8, 9). Genome annotation was completed using Artemis software (10) and a BLAST search.

The complete genome sequence of *S. anginosus* J4211 contains 1,924,513 bp, with a G+C content of 38.99%. The number of predicted coding regions is 1,926, with 13 rRNA genes and 60 tRNA genes. Analysis using the Web-based tool IslandViewer (11) revealed seven genomic islands, including mobile genetic elements and incomplete prophages. The PHAge Search Tool (PHAST), another Web-based program, confirmed the presence of two incomplete prophages (positions 120772 to 153677 and 1362153 to 1402752) (12). There are three clustered regularly interspaced short palindromic repeat (CRISPR) regions (positions 384703 to 385609, 910080 to 911303, and 1299282 to 1299517) in the genome identified by CRISPRFinder (13). The complete capsular polysaccharide (CPS) locus, as mentioned by Tsunashima et al. (14), is present in the genome sequence (positions 1789505 to 1820948). The genome sequence of this *S. anginosus* strain will help us in the future to understand the pathogenic mechanisms of this organism.

### Nucleotide sequence accession number.

The complete genome sequence of *S. anginosus* J4211 has been deposited at GenBank under the accession number no. CP012805. This paper describes the first version of the genome.

## References

[1] Jensen A, Hoshino T, Kilian M 2013 Taxonomy of the *anginosus* group of the genus *Streptococcus* and description of *Streptococcus anginosus* subsp. *whileyi* subsp. nov. and *Streptococcus constellatus* subsp. *viborgensis* subsp. nov. Int J Syst Evol Microbiol 63:2506–2519. doi:10.1099/ijs.0.043232-0. 23223817

[2] Asam D, Spellerberg B 2014 Molecular pathogenicity of *Streptococcus anginosus*. Mol Oral Microbiol 29:145–155. doi:10.1111/omi.12056. 24848553

[3] Whiley RA, Beighton D, Winstanley TG, Fraser HY, Hardie JM 1992 *Streptococcus intermedius*, *Streptococcus constellatus*, and *Streptococcus anginosus* (the *Streptococcus milleri* group): association with different body sites and clinical infections. J Clin Microbiol 30:243–244. 1734062 10.1128/jcm.30.1.243-244.1992 PMC265033

[4] Whiley RA, Fraser H, Hardie JM, Beighton D 1990 Phenotypic differentiation of *Streptococcus intermedius*, *Streptococcus constellatus*, and *Streptococcus anginosus* strains within the “*Streptococcus milleri* group.” J Clin Microbiol 28:1497–1501. 2380375 10.1128/jcm.28.7.1497-1501.1990 PMC267976

[5] Parkins MD, Sibley CD, Surette MG, Rabin HR 2008 The *Streptococcus milleri* group—an unrecognized cause of disease in cystic fibrosis: a case series and literature review. Pediatr Pulmonol 43:490–497. doi:10.1002/ppul.20809. 18383109

[6] Pitcher DG, Saunders NA, Owen RJ 1989 Rapid extraction of bacterial genomic DNA with guanidium thiocyanate. Lett Appl Microbiol 8:151–156. doi:10.1111/j.1472-765X.1989.tb00262.x.

[7] McShan WM, McLaughlin RE, Nordstrand A, Ferretti JJ 1998 Vectors containing streptococcal bacteriophage integrases for site-specific gene insertion. Methods Cell Sci 20:51–57. doi:10.1023/A:1009773309163.

[8] Aziz RK, Bartels D, Best AA, DeJongh M, Disz T, Edwards RA, Formsma K, Gerdes S, Glass EM, Kubal M, Meyer F, Olsen GJ, Olson R, Osterman AL, Overbeek RA, McNeil LK, Paarmann D, Paczian T, Parrello B, Pusch GD, Reich C, Stevens R, Vassieva O, Vonstein V, Wilke A, Zagnitko O 2008 The RAST server: Rapid Annotations using Subsystems Technology. BMC Genomics 9:75. doi:10.1186/1471-2164-9-75. 18261238 PMC2265698

[9] Overbeek R, Olson R, Pusch GD, Olsen GJ, Davis JJ, Disz T, Edwards RA, Gerdes S, Parrello B, Shukla M, Vonstein V, Wattam AR, Xia F, Stevens R 2014 The SEED and the rapid annotation of microbial genomes using subsystems technology (RAST). Nucleic Acids Res 42:D206–D214. doi:10.1093/nar/gkt1226. 24293654 PMC3965101

[10] Rutherford K, Parkhill J, Crook J, Horsnell T, Rice P, Rajandream M-, Barrell B 2000 Artemis: sequence visualization and annotation. Bioinformatics 16:944–945. doi:10.1093/bioinformatics/16.10.944. 11120685

[11] Langille MGI, Brinkman FSL 2009 IslandViewer: an integrated interface for computational identification and visualization of genomic islands. Bioinformatics 25:664–665. doi:10.1093/bioinformatics/btp030. 19151094 PMC2647836

[12] Zhou Y, Liang Y, Lynch KH, Dennis JJ, Wishart DS 2011 PHAST: a fast phage search tool. Nucleic Acids Res 39:W347–W352. doi:10.1093/nar/gkr485. 21672955 PMC3125810

[13] Ibtissem G, Gilles V, Christine P 2007 CRISPRFinder: a Web tool to identify clustered regularly interspaced short palindromic repeats. Nucleic Acids Res 35:W52–W57. 17537822 10.1093/nar/gkm360 PMC1933234

[14] Tsunashima H, Miyake K, Motono M, Iijima S 2012 Organization of the capsule biosynthesis gene locus of the oral streptococcus *Streptococcus anginosus*. J Biosci Bioeng 113:271–278. doi:10.1016/j.jbiosc.2011.10.013. 22100898

